# Protective Effects of Quercetin on Livers from Mice Exposed to Long-Term Cigarette Smoke

**DOI:** 10.1155/2020/2196207

**Published:** 2020-11-15

**Authors:** Pedro A. Machado-Junior, Natália P. S. Araújo, Ana B. F. Souza, Thalles F. Castro, Michel Oliveira, Guilherme P. Costa, Natália A. Matos, Paula M. A. Vieira, André Talvani, Frank S. Bezerra, Sílvia D. Cangussú

**Affiliations:** ^1^Laboratory of Experimental Pathophysiology (LAFEx), Department of Biological Sciences (DECBI), Institute of Exact and Biological Sciences (ICEB), Federal University of Ouro Preto (UFOP), 35400-000 Ouro Preto, MG, Brazil; ^2^Laboratory of Immunobiology of Inflammation (LABIIN), Department of Biological Sciences (DECBI), Institute of Exact and Biological Sciences (ICEB), Federal University of Ouro Preto (UFOP), 35400-000 Ouro Preto, MG, Brazil; ^3^Laboratory of Morphopathology (LM), Department of Biological Sciences (DECBI), Institute of Exact and Biological Sciences (ICEB), Federal University of Ouro Preto (UFOP), 35400-000 Ouro Preto, MG, Brazil

## Abstract

Cigarette smoke is highly toxic, and it can promote increased production of reactive species and inflammatory response and leads to liver diseases. Quercetin is a flavonoid that displays antioxidant and anti-inflammatory activities in liver diseases. This study aimed at evaluating the protective effects of quercetin on livers from mice exposed to long-term cigarette smoke exposure. Male C57BL/6 mice were divided into five groups: control (CG), vehicle (VG), quercetin (QG), cigarette smoke (CSG), quercetin, and cigarette smoke (QCSG). CSG and QCSG were exposed to cigarette smoke for sixty consecutive days; at the end of the exposures, all animals were euthanized. Mice that received quercetin daily and were exposed to cigarette smoke showed a reduced influx of inflammatory cells, oxidative stress, inflammatory reaction, and histopathological changes in the liver, compared to CSG. These results suggest that quercetin may be an effective adjuvant for treating damage to the liver due to cigarette smoke exposure.

## 1. Introduction

Cigarette smoking is considered one of the leading preventable causes of morbidity and mortality representing a risk factor for several diseases such as cardiovascular, chronic obstructive pulmonary, and liver diseases [1, 2]. Smoking kills about 6 million people a year, and it is estimated that this number will reach 8 million by 2030. Cigarette smoke (CS) is a highly toxic complex mixture of components containing more than 5000 compounds that can promote damage to biological tissues [3].

The CS produces reactive oxygen species (ROS) that, at high concentrations, brings about changes in the redox process and induction of inflammatory responses, triggering several comorbidities [4–6]. The exposure to cigarette smoke causes damage to cells and tissues by developing oxidative processes. The installation of oxidative stress can occur directly through the production of ROS or by the activation of the inflammatory process, which, in turn, results in an increased level of radical species [7, 8].

The liver is the biggest gland of the body, which is responsible for processing drugs, alcohol, and other toxins. As an immunologically complex organ, the liver contains multiple types of tissue-resident inflammatory cells, like lymphocytes [9] and Kupffer cells [10]. Therefore, liver diseases represent an important public health problem, and understanding the mechanisms of the pathophysiological process is crucial. The redox imbalance induced by chronic exposure to cigarette smoke produces toxins with direct or indirect toxic effects in the liver, promoting an increase in the number of inflammatory cells, collagen deposition, and apoptosis. Moreover, constituents of cigarette smoke exert immunoregulatory actions and hepatic carcinogens [11].

Previous studies showed that anti-inflammatory and antioxidant activities in natural compounds prevented diseases. In this context, it is worth to highlight the role of quercetin, which is the most abundant flavonol, and it is present in a variety of foodstuffs like fruits and vegetables. Quercetin is present in plants, and it presents many different glycosidic forms (formed by attaching a glycosyl group), which directly reflects on the bioavailability and bioactivity profile of this substance. However, a part of the absorbed quercetin can circulate in the blood as aglycone (the lacking sugar moieties form). Quercetin can act beneficially in several pathologies, especially those that affect the lungs [12–14]. A recent study by our research group showed that quercetin acts as a potent agent in preventing oxidative damage and pulmonary changes [15]. Some studies also extend the effects of quercetin to liver pathologies, exerting a modulating action on inflammation, fibrosis, and redox imbalance through the interaction with a wide spectrum of central molecular targets for cell signaling machines, thus playing a promising role in the treatment of chronic inflammatory liver diseases [16, 17]. However, the ability of quercetin to counteract the deleterious effects of cigarette smoke in the liver has not been elucidated. The goal of this study was to evaluate the effects of quercetin on reducing the redox imbalance and liver inflammation induced by long-term cigarette smoke exposure.

## 2. Material and Methods

### 2.1. Animals

The ethics committee of the Federal University of Ouro Preto (2015/20) approved all animal experiments, and the experiments were performed according to the rules of animal protection and the ethical principles of the Brazilian Society of the Science in Laboratory Animals (SBCAL).

Forty male C57BL/6 mice (12 weeks old) obtained from the Laboratory of Experimental Nutrition (LABNEX) at the Federal University of Ouro Preto (UFOP) were housed under controlled conditions (12 h light/dark, 21°C ± 2°C, 50% ± 10% humidity) with access to food and water ad libitum. The mice were divided into 5 groups (*n* = 8 per group): a control group exposed to ambient air (CG), a group that received 200 *μ*L of vehicle solution containing 50% propylene glycol (Sigma Aldrich, Missouri, USA) and 50% saline (VG) by orogastric gavage, a group that received 10 mg/kg/day of quercetin (Sigma Aldrich, Missouri, USA) diluted in 200 *μ*L of propylene glycol solution (QG), a group exposed to cigarette smoke (CSG), and a group administered with quercetin and exposed to cigarette smoke (QCSG). The administration of quercetin was performed via orogastric gavage 1 h before exposure to cigarette smoke [12, 13].

### 2.2. Cigarette Smoke Exposure Protocol

In a smoking chamber, the mice were exposed to 12 commercial full-flavor filtered Virginia cigarettes (tar: 10 mg, nicotine: 0.9 mg, and carbon monoxide: 10 mg) per day divided into three times daily (morning, afternoon, and night) for 60 consecutive days as previously described [18]. The cigarette smoke (CS) and QCSG animals were initially placed in an inhalation chamber (40cm × 30cm × 25cm) inside an exhaustion chapel. Each cigarette was coupled to a 60 mL plastic syringe through which the smoke was injected inside the inhalation chamber. CSG and QCSG were maintained in the smoke atmosphere for 6 min; then, the cover was removed from the inhalation chamber for 1 min to allow the air to completely exhaust. The procedure was then repeated with the remaining cigarettes. The CG, VG, and QG were subjected to the same conditions, but without exposure to cigarette smoke. At the end of the exposition, the animals were also weighed on a digital scale (Mark®; Série M/Bel Analytical Equipment LTDA, São Paulo, Brazil).

### 2.3. Blood and Liver Collection

The animals were euthanized twenty-four hours after the last exposure to CS by an overdose of ketamine (130 mg/kg) and xylazine (0.3 mg/kg). The blood was collected through the cardiac puncture in polypropylene tubes containing 15 *μ*L of anticoagulants. Subsequently, it was centrifuged at 10.000 rpm for 15 minutes, and the plasma was collected and stored in a freezer (-80°C). Immediately after euthanasia, the right ventricle was perfused with saline to remove blood from the liver; subsequently, two aliquots of liver were separated. The first sample was fixed in 4% neutral buffered formalin, dehydrated, cleared, embedded in paraffin, cut into 4–5 *μ*m sections, and stained with hematoxylin and eosin (H&E) for histopathological analyses. Afterwards, 100 mg of the tissue was placed in polypropylene tubes with 1 mL of phosphate buffer (pH 7.8), and it was homogenized in a tissue homogenizer. Then, the samples were centrifuged at 4°C for 10 minutes and 13.000 rpm (MIKRO 200R; laboratory technology Hettich, Tuttlingen, Germany), and the supernatant was collected and stored at -80°C [15].

### 2.4. Biochemical Measurements

The samples containing 300 *μ*L of plasma were sent to the Pilot Laboratory of Clinical Analysis (Lapac) in Ouro Preto-MG, in order to determine the concentrations of liver function markers, including aspartate aminotransferase (AST; K048-6), alanine aminotransferase (ALT; K049-6), lactate dehydrogenase (LDH; K014-2,) and gamma-glutamyltransferase (GGT-1; K080-2), which were determined by an automatic spectrophotometry in the clinical analyzer Randon Acess Clinical Analyzer, Wiener Lab, model CM-200 (WIENER LAB, Rosario, Argentina) by the enzymatic-colorimetric method using specific kits that were purchased from Bioclin®, Quibasa (MG, Brazil).

### 2.5. Histopathological Analysis

The histopathological analysis was carried out using a light microscope (Leica DFC 300 FX) with a 40x microscopic objective. For histological and morphometric analysis, we obtained twenty randomly chosen images of histological slides that were prepared from the liver sections. These slides were scanned using the Leica Application software. Histological screening focused on the detection and grading of the following abnormalities: inflammatory reaction, sinusoidal congestion, degenerative hepatocyte lesions (hydropic and steatotic), sinusoidal congestion, hyperemia, bleeding focus, intralobular and portal granulomas, fibrosis, and necrosis. The changes in the experimental histopathological parameters present were semiquantified according to a scoring method as (0) absent, (1) mild, when present in approximately less than 33% in the total area of the tissue, (2) moderate, when present between approximately 33% and 66% in the total area of the tissue, and (3) intense, when present in approximately more than 66% in the total area of the tissue [19, 20]. The counting of the number of inflammatory cells present in the hepatic lobes included the resident Kupffer cells. Slides were analyzed using the Leica Q-Win Plus software (Leica Microsystems, Inc., Buffalo Grove, Illinois, USA).

### 2.6. Analysis of Antioxidant Defense and Biomarkers of Oxidative Stress

A hepatic homogenate was used for the analysis of antioxidant defense and oxidative damage. The superoxide dismutase (SOD) activity was measured according to the method described by Marklund and Marklund, based on the enzyme ability to inhibit the autoxidation of pyrogallol [21]. The catalase (CAT) activity was measured using a spectrophotometer according to the method described by Aebi, which is based on the decrease in H_2_O_2_ at an absorbance of 240 nm [22]. The glutathione analysis was determined using an assay adapted from a commercial kit (CS0260, Sigma, St. Louis, MO, USA), and the method is based on the ability to reduce the 5.5′-Dithio Acid-bis-(2-nitrobenzoic) to thio-2-nitrobenzoic acid according to the Griffith assay [23]. In order to calculate the concentration of total glutathione (GSH+GSSG) and oxidized glutathione (GSSG), a standard serial dilution curve was prepared, and the concentration of reduced form (GSH) was calculated from the subtraction of the total glutathione value by the value of oxidized glutathione. In order to measure lipid peroxidation, we used the method described by Buege and Aust [24], which thiobarbituric acid reacts with oxidized lipids and the concentration of TBARS was provided using the molar extinction coefficient, following the law of Lambert Beer. For the determination of the carbonylated protein, a protocol adapted from the methodology described by Reznick and Packer was used [25]. The total protein was measured according to the Lowry method [26].

### 2.7. Immunoenzymatic Assay for Inflammatory Mediators

Interleukin-6 (IL-6), interleukin-10 (IL-10), monocyte chemotactic protein 1 (CCL2), and interferon-*γ* (INF-*γ*) were performed in liver homogenate by the enzyme-linked immunosorbent assay (ELISA) method. The assays were performed using commercial kits (PeproTech, Ribeirão Preto, Brazil), and the antibodies and reagents were prepared according to the instructions from the manufacturer. Immunoenzymatic assays were performed in 96-well plates, on which 100 *μ*L of monoclonal antibody was added to the protein (or peptide) of interest, and the sample were diluted in phosphate-buffered saline (PBS) containing 0.1% fetal bovine serum. After incubation for 12 hours at room temperature, the nonadsorbed antibodies were discarded, and blocking was performed with a solution containing PBS with 1% fetal bovine serum for one hour. Samples of the liver tissue supernatant, which were previously processed and stored at -80°C, were applied in a volume of 50 *μ*L for each well. The reading of the staining intensity was performed in an ELISA reader using a wavelength of 490 nm. The quantification of chemokines and interleukins present in the samples was determined based on the optical density obtained with the peptide standard curve, analyzed by the SOFT Max PRO 4.0 software [27, 28], and the result obtained was a cytokine picogram per milliliter (pg/mL).

### 2.8. Statistical Analysis

The statistical analyses were performed using Prism version 5.00 for Windows 7 (GraphPad Software, San Diego, CA USA). The normal distribution of each variable was assessed using the Kolmogorov-Smirnov test. The normally distributed data were analyzed with one-way ANOVA followed by Tukey's posttest, and the data are expressed as mean ± standarderrorofthemean (SEM). For nonparametric data, we used the Kruskal Wallis test followed by Dunn's posttest, and the data are expressed as the median, minimum, and maximum values. In both cases, the difference was considered significant when the *p* value was <0.05.

## 3. Results

### 3.1. Hepatic Function Markers and Body Weight

The animals from the five experimental groups were evaluated to investigate the effects of cigarette smoke on liver damage biomarkers. It can be seen from Table 1 that a higher level of ALT (ANOVA, *p* = 0.0071) in the animals was exposed to long-term cigarette smoke (CSG) when compared with the control groups (*p* < 0.05). However, there were no significant differences between the CSG and QCSG regarding the markers of the liver function (Table 1).

There was a decrease of the final body weight (ANOVA, *p* < 0.0001) of the CSG compared to CG and VG (*p* = 0.001). However, there was no difference with respect to the final body weight between CSG and QCSG (*p* > 0.05) (Table 1).

### 3.2. Histopathological Aspects

**Figure 1:**
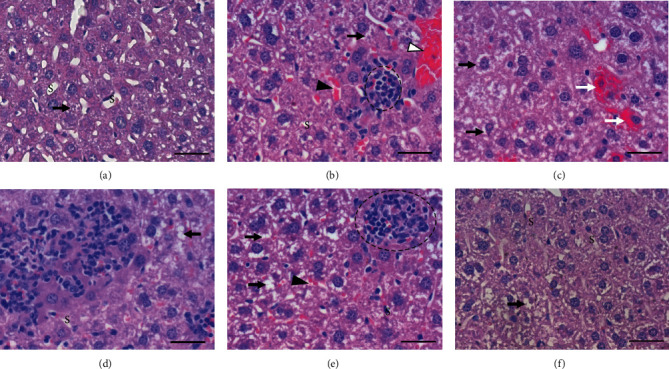
Histopathological aspects of the liver. Photomicrographs of liver sections stained with hematoxylin and eosin. Bar = 50*μ*m, 400x magnification. (a) Representative image of groups exposed to ambient air (CG, VG, QG). (b)–(e) Representative images of the lesions found in the group exposed to cigarette smoke (CSG). (f) Representative image of the group pretreated with quercetin and exposed to cigarette smoke (QCSG). (a) Preserved liver parenchyma, presence of hydropic degeneration (black arrow), and preserved sinusoid capillaries (S). (b) Hydropic degeneration (black arrow), congestion of sinusoid (black arrowhead), presence of granuloma (dotted circle), and hyperplasia (white arrowhead). (c) Hydropic degeneration (black arrow) and bleeding focus (white arrow). (d) Inflammatory cell infiltration and hydropic degeneration (black arrow). (e) Hydropic degeneration (black arrow), congestion of sinusoid (black arrowhead), and presence of granuloma (dotted circle). (f) Preserved hepatic parenchyma notes the hydropic degeneration (black arrow) and well-preserved sinusoid capillaries (S).

The semiquantitative analyzes were performed for the presence and intensity of hepatic lesions of mice from the experimental groups. The histopathological evaluation revealed that the control group (CG) had almost completely preserved liver histology (Figure 1(a), Tables 2 and 3). Exposure to cigarette smoke (CSG) resulted in changes in the liver parenchyma such as inflammatory reaction, sinusoidal congestion, hyperemia, granulomatous reaction, and bleeding focus (Figures 1(b)–(e), Tables 2 and 3). The quercetin administration (QCSG) was able to improve the effects of long-term cigarette smoke exposure, with decreasing or the disappearing of injuries (Figure 1(f), Tables 2 and 3). Hydropic degeneration was observed in all groups; however, there were no significant differences (Figure 1, Tables 2 and 3).

The semiquantitative analysis showed higher frequency of sinusoidal congestion/hyperemia in livers of mice exposed to long-term cigarette smoke (75%) than all groups (CG-0%, VG-12.5%, QG-12.5%, QCSG-12.5%) (Table 2). Moreover, the quercetin administration associated to CS (QCSG) provoked an increase in the severity of this injury (12.5%) compared to CSG (*p* = 0.0008) (Table 3). In the CSG, this lesion ranged from absent (25%), moderate (25%), to intense (50%) in severity (Table 3). While in the other groups, the sinusoidal congestion/hyperemia ranged only from absent (CG: 100%, VG: 87.5%, QG: 87.5%, QCSG: 87.5%) to mild intensity (VG: 12.5%, QG: 12.5%, QCSG: 12.5%) (Table 3).

The bleeding focus and granulomatous reactions were observed only in animals exposed to long-term cigarette smoke (CSG) (25% and 75%, respectively) (Figures 1(b), 1(c), and 1(e) and Table 2).

The inflammatory reaction was more frequent in the group exposed to CS (CSG) (87.5%) than VG (25%) and QG (12.5%) (Figures 1(b), 1(d), and 1(e) and Table 2). There was not any inflammatory reaction in the control group (CG: 0%); in these animals, only the resident inflammatory cells were observed (Figure 1(a), Table 2). The quercetin administration associated to cigarette smoke (QCSG) decreased the frequency of the inflammatory reaction to 12.5% of the animals (*p* = 0.0003) (Table 2), i.e., the inflammatory reaction reduced to values similar to those of the VG and QG. Moreover, the inflammatory reaction was more intense in CSG, presenting a mild range from (37.5%) to moderate (37.5%) to intense, while the other groups presented only mild intensity, when observed (VG: 25%, QG: 12.5%, QCSG: 12.5%) (Table 3).

Moreover, hepatic fibrosis and necrosis were not observed in the animals studied (Table 2).

**Figure 2:**
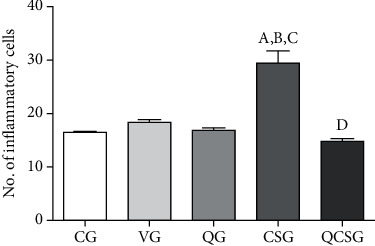
Number of inflammatory nucleus into the hepatic parenchyma of C57BL/6 mice. CG: control group; VG: vehicle group; QG: quercetin group; CSG: cigarette smoke group; QCSG: quercetin and cigarette smoke group. ^a^ represents a significant difference between groups when compared to CG. ^b^ represents a significant difference between groups when compared to VG. ^c^ represents a significant difference between groups when compared to QG. ^d^ represents a significant difference between groups when compared to CSG. Data were expressed as mean ± standarderrorofthemean and were analyzed by one-way ANOVA followed by Tukey's posttest, *n* = 8 animals per group (*p* < 0.05).

The number of inflammatory cells to the liver was greater (ANOVA, *p* < 0.0001) in animals exposed to cigarette smoke (CSG) (29.43 ± 2.24) compared to control groups (CG: 16.51 ± 0.11, VG: 18.39 ± 0.41, QG: 16.83 ± 0.49) (*p* = 0.001). Pretreatment with quercetin (14.82 ± 0.52) resulted in a lower number of inflammatory cells in the liver when compared to CSG (*p* = 0.001) and presented values similar to those of the control group (Figure 2).

### 3.3. Antioxidant Defense and Oxidative Stress Biomarkers in the Liver

Cigarette smoke exposure promoted an increase of the SOD activity (ANOVA, *p* = 0.0009) compared to CG, VG, and PG (*p* < 0.05), which was attenuated by pretreatment with quercetin (*p* = 0.001). The catalase activity was lower (ANOVA, *p* = 0.0019) in the CSG and QCSG groups compared to the control (*p* = 0.01). The GSH/GSSG ratio was lower (Kruskal Wallis, *p* = 0.0158) in the CSG than QC (*p* < 0.05), and the administration of quercetin increased the GSH/GSSG ratio compared to CSG (*p* < 0.05). The damage to liver parenchyma was evaluated based on levels of TBARS (ANOVA, *p* < 0.0001); as a result, the lipid oxidation was higher in the group exposed to cigarette smoke compared to CG, VG, and QG (*p* = 0.001), and the lipid oxidation was also attenuated by pretreatment with quercetin (*p* = 0.001) (Table 4).

### 3.4. Inflammatory Mediators' Levels in the Liver

Inflammatory markers IL-6, IL-10, INF-*γ*, and CCL2 were measured in order to assess the inflammatory status of the liver. The animals exposed to cigarette smoke for sixty days showed higher levels of INF-*γ* (ANOVA, *p* = 0.0002) and IL-10 (ANOVA, *p* = 0.0005) in liver homogenate when compared with CG, VG, and QG (*p* = 0.01). For both markers, pretreatment with quercetin led to a decrease in cytokine levels compared to CSG (*p* < 0.05). The levels of CCL2 chemokine (ANOVA, *p* < 0.0001) in the liver of the group exposed to smoke were lower than the control group (*p* = 0.001), and the administration of quercetin promoted an increase in the levels of this marker compared to CSG (*p* = 0.001). For the analysis of IL-6, no statistical difference was observed between the experimental groups (ANOVA, *p* = 0.65) (Table 5).

## 4. Discussion

In this study, we investigated the antioxidant effect of quercetin against the oxidative stress, inflammation, and changes in liver morphology induced by cigarette smoke. We observed morphological changes and redox imbalance in the livers of mice exposed to long term-cigarette smoke, strengthening the deleterious effect of cigarette smoke on the liver parenchyma, corroborating previous studies [29–31]. A study conducted by Ogenyi et al. revealed an adverse effect of passive cigarette smoke on the liver morphology and biochemistry of Wistar rats [31]. Moreover, a study conducted by Adedayo et al. reported that the smoke extract of tobacco nicotine has adverse and compromising effects on the liver of male Sprague-Dawley rats [30].

Liver diseases represent an important focus of attention in view of the multiple functions of the liver in the body. Use of herbal drugs in the treatment of liver diseases has a long tradition [17]. In this context, quercetin, the most abundant flavonoid in nature, has been found to exhibit a potent antioxidant capacity and the ability to counteract several insults to the liver. Kalantari et al. demonstrated that quercetin is effective for the prevention of tert-butyl hydroperoxide-induced hepatic damage in mice [32]. Similarly, Lee et al. reported that quercetin prevents liver inflammatory injury, induced in rats by ethanol extracts from tartary buckwheat [33]. However, to our knowledge, this is the first study that examines the antioxidant effect of quercetin against deleterious changes in liver morphology and in the oxidative stress induced by cigarette smoke.

We observed that there was a higher level of ALT in the animals exposed to long-term cigarette smoke when compared with the control group. ALT is one of the most used biomarkers for measuring liver injury, and several studies with cigarette smoke use this biomarker to estimate the harmful effects of smoke on the liver tissue [9, 34, 35]. Studies describe the role of quercetin in reducing liver damage, in face of an injury-causing agent, by determining ALT levels [36–38]. However, despite the findings of the current study showing that quercetin combats ROS, inflammation, and helps with antioxidant defense, there was no statistical difference between the ALT levels between CSG and QCSG.

Previous studies have demonstrated a reduction in the body weight in animals exposed to cigarette smoke [1, 18, 39]. A study by our research group correlated this reduction in the body weight with inflammation and subsequent secretion of TNF-*α*, in addition to food satiety in animals exposed to cigarette smoke [18]. Here, our results corroborate with these studies, where a decrease in the body weight was observed in the animals exposed to CS, perhaps as a consequence of the inflammatory process in the liver tissue. Pretreatment with quercetin was not able to reverse this parameter. Our results are in agreement with other studies that did not observe changes in the body weight with low concentration of quercetin [40–42].

We observed that the quercetin administration was able to improve the effects of long-term cigarette smoke exposure, with injury decreases resulting from this exposure, such as inflammatory reaction, sinusoidal congestion, hyperemia, granulomatous reaction, and bleeding focus. Moreover, we observed a significant increase in the number of inflammatory cells into the liver in response to cigarette smoke exposure that may be directly related to the time of exposure to cigarette smoke, which was long. Several studies showed a relationship between cigarette smoke and a greater influx of total leukocytes, either by the formation of reactive oxygen species or by the presence of nicotine [43–45]. The quercetin reduced the number of inflammatory cells to the liver in animals exposed to long-term cigarette smoke, supporting the cytoprotective and anti-inflammatory effects of quercetin [46]. A similar result showed by Barros et al. found evidence of the hepatoprotective quercetin activity in acute paracetamol toxicity, as shown by a reduction in necrosis and inflammation [47].

Previous data showed that the cigarette smoke exposure promoted changes in the antioxidant enzyme activity and oxidative damage in the liver of mice and rats [48, 49]. We evaluated the redox imbalance by measuring oxidative damage, and our results showed that the cigarette smoke significantly increased the activity of the SOD, and the quercetin administration restored their activity to control levels. These results prove the ability of quercetin to combat and neutralize reactive oxygen species (ROS), preventing oxidative stress according to Dias et al. [50]. These authors found that quercetin, by scavenging ROS, prevents the elevation of the SOD activity in the liver of streptozotocin-induced diabetic rats. The increase in the SOD activity in the liver tissue caused by cigarette smoke has also been described [51, 52] in rats.

GSH is a key player in the maintenance of the cellular redox status and exists primarily in two redox forms, reduced (GSH) and oxidized (GSSG) [53]. The GSH/GSSG ratio can serve as a good indicator of the cellular redox state [54]. Our results indicated that CS reduced the GSH/GSSG ratio, while quercetin, in turn, was able to restore the GSH/GSSG ratio. This result possibly indicates a greater activity of GPx in the group exposed to cigarette smoke and a lower activity of this enzyme in group pretreated with quercetin and exposed to cigarette smoke. Therefore, we believe that quercetin will neutralize ROS instead of GPx, thus maintaining the redox status of the cells. Our data corroborate with the results from a previous study by Olayinka et al. and Chen, 2010, which reported protective effects of quercetin, regarding the ability of quercetin to increase the GSH/GSSG ratio in liver injury induced by procarbazine and ethanol, respectively [55, 56]. Therefore, an increase in the antioxidant system and consequent reduction of ROS induced by the quercetin administration observed in the present study may be a main factor for protection of the liver against injury caused by CS exposure.

In our experimental model, the CAT activity decreased in CSG compared to that in control groups, and the quercetin administration was unable to regulate the activity of this enzyme. Similarly, a series of studies in the lung and liver reported a decrease of the catalase activity in models that promote an increase in ROS [18, 57–59]. A depletion of catalase reserves promoted by a persistent increase in production of reactive oxygen species may have occurred. In the other hand, the liver has one of the highest organ contents of GSH [60, 61]. The glutathione system is expected to be more active in neutralizing ROS production after exposure to cigarette smoke when compared to other components of the antioxidant system, such as the CAT activity. Despite the decreased CAT activity, quercetin showed a strong antioxidant activity by maintaining oxidative balance. According to Xu et al., the antioxidant activity of quercetin is mainly manifested through its effect on glutathione [62].

In this study, the long-term cigarette smoke significantly increased the TBARS levels that were restored by the quercetin administration. These results are in accordance with previous studies that evaluated the effect of cigarette smoke on the liver [34, 52, 63]. Some studies in liver damage have also demonstrated that quercetin modulates lipid and lipoprotein metabolism damage caused by oxidative stress inducing agents other than cigarette smoke [32]. Our results demonstrate that quercetin is effective in decreasing lipid peroxidation and in preventing oxidative damage in the liver parenchyma of mice exposed to long-term cigarette smoke.

We observed a decrease of CCL2 and IFN-*γ*, as well as an increase of IL-10 in the C57BL/6 mice exposed to long-term CS. Regarding the levels of IL-6 in the hepatic homogenate, there were no changes observed between the experimental groups. As the primary injury of cigarette smoke occurs in the lung, it is expected that these inflammatory markers are more active in this organ, compared to other organs such as the liver [18]. The IL-10 increase in the liver tissue of mice exposed to CS may be a response to the influx of inflammatory cells to that organ, and the concomitant decrease in proinflammatory mediators, such as CCL2 and INF-*γ*, may be a consequence of the IL-10 which regulates the proinflammatory activity of these mediators, and it has been described in immunologic studies in several organs [44, 64, 65]. Studies describe the regulation of IL-10 over other cytokines and chemokines, and, depending on the inflammatory profile of the region with injury caused by oxidant agents, it exerts a modulation on INF-*γ* and CCL2 [66, 67].

Our data demonstrated that the pretreatment with quercetin restored the levels of inflammatory markers to the levels of the control group and are in agreement with those of Chen, who demonstrated the role of quercetin in restoring cytokine and chemokine levels in the liver tissue exposed to ethanol [56]. Other works have also described in several organs, the role of quercetin in modulating inflammatory response through the induction or repression of pro or anti-inflammatory cytokines [13, 18, 33, 46, 68]. However, this is the first study that examines the antioxidative and anti-inflammatory properties of quercetin against deleterious changes in liver morphology and in the oxidative damage induced by cigarette smoke.

In summary, these results suggest that quercetin has a promising role as an auxiliary pharmacological tool in the treatment of damage to the liver due to the exposure to cigarette smoke.

## Figures and Tables

**Table 1:** Serum biochemical parameters and body weight of C57BL/6 mice.

	CG	VG	QG	CSG	QCSG
ALT (U/L)	12.41 ± 1.17	12.56 ± 1.90	11.56 ± 1.72	24.50 ± 4.75^a,b,c^	18.80 ± 2.27
AST (U/L)	88.36 ± 5.45	96.85 ± 10.4	88.26 ± 5.49	83.80 ± 6.93	83.80 ± 4.40
GGT-1 (U/L)	2.46 ± 0.82	2.81 ± 0.61	4.87 ± 0.42	3.38 ± 0.56	2.98 ± 0.50
LDH (U/L)	386.2 ± 31.1	412.2 ± 23.5	356.4 ± 18.8	383.5 ± 26.6	389.4 ± 45.0
Body weight (g)	27.78 ± 0.38	26.83 ± 0.31	25.60 ± 0.97	24.11 ± 0.65^a,b^	23.47 ± 0.34^a,b^

CG: control group; VG: vehicle group; QG: quercetin group; CSG: cigarette smoke group; QCSG: quercetin and cigarette smoke group, ALT: alanine aminotransferase; AST: aspartate aminotransferase; GGT-1: gamma-glutamyltransferase; LDH: lactate dehydrogenase. ^a^ represents a significant difference between groups when compared to CG; ^b^ represents a significant difference between groups when compared to VG; ^c^ represents a significant difference between groups when compared to QG. ALT, AST, GGT-1, LDH, and body weight were expressed as mean ± standarderrorofthemean and were analyzed by one-way ANOVA followed by Tukey's posttest, *n* = 5 − 8 animals per group (*p* < 0.05).

**Table 2:** Frequency of histopathological changes in the liver of C57BL/6 mice in the experimental groups.

Lesions	CG *N* = 8 (%)	VG *N* = 8 (%)	QG *N* = 8 (%)	CSG *N* = 8 (%)	QCSG *N* = 8 (%)
Hydropic degeneration	8 (100)	8 (100)	8 (100)	8 (100)	8 (100)
Sinusoidal congestion/hyperemia	0 (0.0)	1 (12.5)	1 (12.5)	6 (75.0)	1 (12.5)
Bleeding focus	0 (0.0)	0 (0.0)	0 (0.0)	2 (25.0)	0 (0.0)
Granulomas	0 (0.0)	0 (0.0)	0 (0.0)	6 (75.0)	0 (0.0)
Inflammatory reaction	0 (0.0)	2 (25.0)	1 (12.5)	7 (87.5)	1 (12.5)
Fibrosis	0 (0.0)	0 (0.0)	0 (0.0)	0 (0.0)	0 (0.0)
Necrosis	0 (0.0)	0 (0.0)	0 (0.0)	0 (0.0)	0 (0.0)

CG: control group; VG: vehicle group; QG: quercetin group; CSG: cigarette smoke group; QCSG: quercetin and cigarette smoke group.

**Table 3:** The semiquantitative score values (score minimum-score maximum) of the main histological lesions found in the livers of experimental animals.

Lesions	CG	VG	QG	CSG	QCSG
Hydropic degeneration	1 (1-2)	1 (1-2)	1 (1-2)	3 (1-3)	1 (1-3)
Sinusoidal congestion/hyperemia	0	0 (0-1)	0 (0-1)	2 (0-3)^abc^	0 (0-1)^d^
Inflammatory reaction	0	0 (0-1)	0 (0-1)	1 (0-3)^abc^	0 (0-1)^d^

CG: control group; VG: vehicle group; QG: quercetin group; CSG: cigarette smoke group; QCSG: quercetin and cigarette smoke group. ^a^ represents a significant difference between CSG when compared to CG; ^b^ represents a significant difference between CSG when compared to VG; ^c^ represents a significant difference between CSG when compared to QG; ^d^ represents a significant difference between groups when compared to CSG. Sinusoidal congestion/hyperemia (*p* = 0.0008). Inflammation reaction (*p* = 0.0003). The data were analyzed by Kruskal-Wallis followed by Dunn's posttest (*n* = 8 animals per group).

**Table 4:** Antioxidant defense and oxidative stress biomarkers in liver homogenate.

	CG	VG	QG	CSG	QCSG
SOD (U/mg protein)	90.74 ± 4.72	88.01 ± 5.0	93.26 ± 5.42	115.4 ± 5.85^a,b,c^	81.42 ± 5.36^d^
CAT (U/mg protein)	0.28 ± 0.02	0.20 ± 0.03	0.22 ± 0.01	0.13 ± 0.03^a^	0.14 ± 0.02^a^
GSH/GSSG ratio	7.21 (2.59; 13.30)	5.84 (4.11; 6.85)	6.05 (5.24; 9.04)	0.41 (0.34; 1.40)^c^	6.61 (3.19; 10.64)^d^
TBARS (nmol/mg protein)	0.34 ± 0.02	0.24 ± 0.02	0.31 ± 0.02	2.07 ± 0.50^a,b,c^	0.27 ± 0.04^d^
Protein carbonyl (nmol/ng protein)	12.73 ± 1.28	13.13 ± 1.90	16.07 ± 2.05	16.18 ± 0.93	14.12 ± 1.58

CG: control group; VG: vehicle group; QG: quercetin group; CSG: cigarette smoke group; QCSG: quercetin and cigarette smoke group, SOD: superoxide dismutase; CAT: catalase; GSH: glutathione sulfide; GSSG: oxidized glutathione; TBARS: thiobarbituric acid reactive substances. ^a^ represents a significant difference between groups when compared to CG; ^b^ represents a significant difference between groups when compared to VG; ^c^ represents a significant difference between groups when compared to QG; ^d^ represents a significant difference between groups when compared to CSG. SOD, CAT, TBARS, and protein carbonyl were expressed as mean ± standarderrorofthemean and were analyzed by one-way ANOVA followed by Tukey's posttest, *n* = 5 − 8 animals per group (*p* < 0.05). The GSH/GSSG ratio data were expressed in median, minimum, and maximum value and were analyzed by Kruskal-Wallis followed by Dunn's posttest that was used, *n* = 5 − 8 animals per group (*p* < 0.05).

**Table 5:** Inflammatory markers in the liver of experimental groups.

	CG	VG	QG	CSG	QCSG
CCL2 (pg/mL)	4025.3 ± 137.6	3279.4 ± 250.9	3153.2 ± 134.4	1852.4 ± 303.7^a,b,c^	3693.6 ± 175.9^d^
INF-*γ* (pg/mL)	537.56 ± 11.85	513.25 ± 42.14	559.05 ± 42.42	324.62 ± 21.59^a,b,c^	519.28 ± 24.11^d^
IL-10 (pg/mL)	1466.3 ± 99.79	1529.2 ± 149.5	1471.6 ± 43.6	2342.3 ± 218.1^a,b,c^	1698.6 ± 57.5^d^
IL-6 (pg/mL)	1711.2 ± 57.7	1712.0 ± 49.6	1688.1 ± 48.1	1780.6 ± 57.3	1668.7 ± 56.1

CG: control group; VG: vehicle group; QG: quercetin group; CSG: cigarette smoke group; QCSG: quercetin and cigarette smoke group, CCL2 or MCP1: monocyte chemotactic protein 1; INF-*γ*: interferon-*γ*; IL-10: interleukin 10; IL-6: interleukin 6. ^a^ represents a significant difference between groups when compared to CG; ^b^ represents a significant difference between groups when compared to VG; ^c^ represents a significant difference between groups when compared to QG; ^d^ represents a significant difference between groups when compared to CSG. Data were expressed as mean ± standarderrorofthemean and were analyzed by one-way ANOVA followed by Tukey's posttest, *n* = 8 animals per group (*p* < 0.05).

## Data Availability

I declare that data related to the manuscript entitled “Protective effects of quercetin on livers from mice exposed to long-term cigarette smoke” are available for publicy.
